# Network analysis of anxiety, depression, stress, and sleep disturbances in healthcare workers

**DOI:** 10.3389/fpsyt.2026.1726751

**Published:** 2026-06-04

**Authors:** Jinni Xu, Jiaqi Wang, Zhifeng Lin, Danna Wang, Shun Liu, Yifei Lin, Qingquan Chen, Liangming Wang, Chunmei Fan, Lufeng Chen

**Affiliations:** 1 The Second Affiliated Hospital of Fujian Medical University, Quanzhou, Fujian, China; 2 Fujian Medical University, Fuzhou, China; 3 Key Laboratory of Sleep Medicine (The Second Affiliated Hospital, Fujian Medical University), Fujian Province University, Quanzhou, Fujian, China

**Keywords:** anxiety, depression, medical staff, network-analysis, PSQI, sleep disturbances, stress

## Abstract

**Background:**

This study employed a network analysis approach to examine the interrelationships between anxiety, depression, stress, and sleep disturbances among healthcare workers, providing a symptom-level perspective on these prevalent mental health concerns.

**Methods:**

This cross-sectional survey was conducted among healthcare workers from medical institutions in Fujian Province, China. Data were collected using the Depression-Anxiety-Stress Scale-21 (DASS-21) and the Pittsburgh Sleep Quality Index (PSQI). After item redundancy screening, Gaussian graphical models with EBICglasso regularization were estimated in R. Nodes represented DASS-21 items and PSQI components, and edges represented regularized partial correlations after conditioning on all other nodes. Network accuracy and stability were assessed using bootstrap procedures, and subgroup differences by gender, length of employment, and education level were examined using the Network Comparison Test.

**Results:**

This study included 1, 335 participants. Network analysis revealed that fear without cause was the most central symptom, with ‘panic’ and ‘overreaction’ also identified as key symptoms within the network. Bridging symptoms, such as overreaction, persistent sadness, were found to show high bridge strength between anxiety, depression, and stress. The network of comorbid symptoms and disturbances highlighted daytime dysfunction and sleep disturbances as central sleep-related nodes associated with psychological symptoms. No significant differences were observed in the network characteristics between the gender and length of employment subgroups in the subgroup analysis; however, differences were noted in the network characteristics based on education level. Persistent sadness and lack of enjoyment were also identified as key factors contributing to the overall psychological symptom network.

**Conclusion:**

This study identified key symptom-level associations among anxiety, depression, stress, and sleep disturbances in healthcare workers. Fear without cause, panic, overreaction, persistent sadness, lack of enjoyment, daytime dysfunction, and sleep disturbances may be useful candidate targets for future longitudinal and intervention studies. These findings should be interpreted as cross-sectional associations rather than causal relationships.

## 1 Introduction

Mental health issues, including anxiety, depression, and stress, are a significant concern within the healthcare sector. Studies have shown that the likelihood of experiencing mental health problems is higher among employees in high-stress occupations, and patients often exhibit emotional tension, depression, and diminished interest in daily activities (1). The work of healthcare professionals is characterized by high intensity and risk, which increases their likelihood of developing mental health problems, particularly during the COVID-19 pandemic, when medical workers face heightened occupational and psychological risks (2). Statistically, anxiety and depression are the most prevalent mental health conditions among healthcare workers, with overall prevalence rates of 33% and 28%, respectively (3). According to statistics, up to 1 trillion dollars are spent globally each year on treating select mental disorders, such as depression and anxiety, resulting in a significant economic burden (4). The declining mental health of healthcare workers not only impacts their well-being but also compromises the quality of care they provide, leading to adverse social consequences and further burdens (5). Currently, mental health issues are primarily addressed through psychopharmacotherapy, psychotherapy, and cognitive interventions. However, additional clinical studies are needed to evaluate the efficacy of these psychological treatments and inform their application (6).

Disturbances primarily encompass disorders such as insomnia, sleep fragmentation, and circadian rhythm disturbances, all of which can lead to unhealthy sleep durations and poor sleep quality. They may negatively impact brain health (7). Dan Qiu et al. reported that the prevalence of sleep disturbances among Chinese healthcare workers was 39.2% (8), which is significantly higher than that in the general population. In addition, a systematic review and meta-analysis showed that, during the COVID-19 pandemic, the prevalence of sleep disturbances in this population rose to 45.1% (9). Healthcare workers often face unavoidable nighttime workloads, which can significantly disrupt their sleep patterns. Statistics show that approximately 50% of hospital night shift workers experience at least one adverse sleep outcome (10). A study on sleep deprivation found that night shift work or 24-hour shifts are associated with mild to moderate episodes of depression, anxiety, and stress symptoms (11). However, while disturbances have been linked to mental health issues, the specific effects of sleep duration, sleep quality, and the factors influencing sleep have yet to be analyzed separately.

Network analysis is an effective method for analyzing and understanding psychiatric disorders by breaking down the various symptoms of a disorder and treating them as distinct nodes that interact with one another. These nodes are connected by edges representing conditional associations (12). Node centrality statistics can be used to evaluate the connectedness of these nodes and identify symptoms that are strongly associated with other symptoms in the network. In cross-sectional networks, however, centrality should be interpreted as connectedness rather than evidence of causal influence. These core symptoms often play a significant role in the onset or maintenance of a psychiatric disorder (13). The theoretical underpinnings of network analysis are rooted in the network theory of psychopathology, which posits that mental disorders arise from direct interactions and feedback loops among symptoms, rather than being driven by a single, unobserved underlying cause (14). This perspective emphasizes the dynamic interplay among symptoms and highlights that network analysis can offer novel insights into the mechanisms underlying comorbidity (15). This perspective is particularly crucial for elucidating the comorbidity mechanisms of anxiety, depression, stress, and disturbances among healthcare workers: these mental health issues often manifest as complex interconnections among symptoms, and network theory can precisely reveal the underlying logic of such multi-symptom interactions instead of simplistically attributing them to a single latent cause. Therefore, network analysis can help elucidate the relationships between different mental health issues and comorbid disturbances, providing information about central and bridge symptoms that may inform future longitudinal and intervention studies.

Multiple studies have shown that insomnia often co-occurs with psychological problems such as anxiety and depression, and these three conditions are closely interconnected (16–18). However, a critical review of existing literature reveals significant gaps in the current research landscape. Previous network analysis studies have predominantly focused on examining the relationship between anxiety and depression among medical staff during the COVID-19 pandemic. For instance, Ouyang et al. conducted a network analysis of post-traumatic stress, depression, and anxiety symptoms among frontline healthcare workers, but sleep-related symptoms were only briefly mentioned as bridge symptoms without being systematically integrated into the core network model (19). Similarly, recent Chinese studies on healthcare workers have utilized network analysis to map anxiety and depression symptom networks, identifying central symptoms such as “inability to relax” and “excessive worry” as key intervention targets. While Zhao et al. examined comorbid insomnia and depressive symptoms among psychiatric practitioners, their study did not include stress symptoms and examined sleep primarily within a depression context rather than as an independent construct alongside anxiety and depression (20).

The present study makes three incremental contributions. First, it integrates stress symptoms into a unified network model with anxiety, depression, and sleep disturbances among healthcare workers. Prior network analyses have often paid less attention to stress, focusing on anxiety-depression comorbidity or treating sleep as a peripheral outcome rather than examining it as a separate component. Second, it systematically analyzes sleep symptoms as distinct nodes, rather than as aggregated comorbidities. This allows precise identification of how specific sleep disturbances connect to anxiety, depression, and stress. Third, it identifies core and bridge symptoms across the full network, providing additional clinical insights for future intervention studies. Relatively few studies have systematically identified such bridge symptoms across all four domains in healthcare workers. The findings will help practitioners address psychological and sleep disturbances simultaneously rather than in isolation. In summary, this study extends previous work by including disturbances as central nodes alongside anxiety, depression, and stress in a unified network model for healthcare workers, revealing the complex interrelationships among these conditions.

## 2 Methods

### 2.1 Study design

This cross-sectional survey was conducted in medical institutions across Fujian Province, with the study period spanning from June 25th to July 14th. The standardized psychological assessment tool DASS-21, in conjunction with the Pittsburgh Sleep Quality Index (PSQI), was used as the primary assessment instrument. Based on network analysis, we developed a multidimensional symptom network model to systematically analyze symptom clusters, central symptoms, co-occurrence patterns, and pairwise conditional associations among nodes. During the validation phase, the bootnet bootstrap method was employed to assess the stability and accuracy of the network structure. Additionally, edge-weight confidence intervals were calculated to verify estimation accuracy, and network differences across various symptom networks were examined through the comparison of centrality indicators.

### 2.2 Participants

This study focuses on the healthcare practitioner population and employs a two-stage mixed sampling design. In Stage 1, the target population was divided into distinct, non-overlapping strata based on healthcare specialty, with practitioners grouped by their specific roles (e.g., physicians, nurses, pharmacists, allied health professionals). In Stage 2, systematic random sampling was conducted within each specialty stratum by selecting participants at fixed intervals (e.g., every kth practitioner) from a randomly ordered list of practitioners within each group. All sampling was limited to healthcare institutions and registered practitioners within Fujian Province, China. Data were collected using the online research platform “Questionnaire Star, “ a digital tool that aligns with the methodological frameworks established in the existing literature (21, 22). The questionnaires were distributed and collected via mainstream instant messaging applications (QQ/WeChat). The response rate for the questionnaire was 95.29%. To ensure the validity of the research data, we implemented a multi-dimensional quality control system: (1) established reasonable response time thresholds (6–10 minutes) to filter out atypical time records; (2) enabled IP address uniqueness checks to prevent duplicate responses; (3) excluded questionnaires with more than 5% missing data; and (4) employed a logical consistency detection module to identify and remove contradictory responses. For the selected participant questionnaires (i.e., those with missing data ≤5%), any remaining missing values were handled using complete case analysis (CCA), meaning that only cases with complete data for a specific variable were included in subsequent statistical analyses.

In empirical studies, sample size calculation is typically conducted through efficacy analysis. Previous research has demonstrated (23) that the minimum sample size required to detect the true effect size can be estimated by simulating data based on preset network topology parameters using the netSimulator tool. Given that the symptom co-occurrence network involves multi-dimensional node attributes and dynamic associations, it is necessary to expand the sample size to ensure the reproducibility of the network structure. Therefore, we developed a simulation model that integrates co-morbid symptom clusters with the disturbance network structure. The results of this model are presented in **Supplementary Figure 1**.

This study successfully collected 1, 335 samples that met the inclusion criteria. Standardized data collection protocols were followed throughout the study, and an encrypted coding system was employed to implement a double anonymity mechanism, ensuring participant privacy and the reliability of data traceability. All participants provided informed consent before participating in the study, and the research protocol was reviewed and approved by the institutional ethics committee.

### 2.3 Measurement

#### 2.3.1 Depression-anxiety-stress scale

We used the Short Form of the Depression, Anxiety, and Stress Scale (DASS-21) to assess the levels of depression, anxiety, and stress in the population. The DASS-21 simultaneously assesses depression, anxiety, and stress, whereas the PHQ-9 focuses solely on depressive symptoms; thus, the DASS-21 is more appropriate for examining the interactive network among anxiety, depression, and stress. Moreover, the Chinese version of the DASS-21 has been validated in several studies involving healthcare workers, university students, and other populations. Li-Chen Jiang et al. reported that the Chinese DASS-21 demonstrates excellent internal consistency among hospital staff (Cronbach’s α = 0.91 for the total scale) and exhibits satisfactory discriminant validity (24). It has also been widely utilized for scientific research and clinical screening. Originally developed by Lovibond et al. in 2007, the scale used in the present study is the revised version by Assault and Lifu et al. The scale consists of 21 items, divided into three dimensions: depression, anxiety, and stress. Each item is rated on a 4-point scale, ranging from 0 (not at all) to 3 (almost every day). The score for each item is calculated by multiplying the response by two, with a maximum possible score of 42 for each dimension. Subjects are classified as having “clinically significant” symptoms if they score ≥10 for depression, ≥8 for anxiety, and ≥15 for stress (25).

#### 2.3.2 Pittsburgh sleep quality index

The Pittsburgh Sleep Quality Index (PSQI) was employed as a screening tool for sleep disturbances in this study. Developed by Buysse et al. at the University of Pittsburgh in 1989, the PSQI is a standardized self-report instrument widely employed in both clinical practice and research. The scale comprehensively evaluates sleep status across seven components: sleep quality, sleep latency, sleep duration, sleep efficiency, sleep disturbances, use of sleep medication, and daytime dysfunction (26). As a standardized self-assessment scale, its 19-item measurement system demonstrates strong reliability and validity (Cronbach’s alpha 0.87) (27). Its Chinese version has been validated in multiple Chinese samples; Niu S et al. confirmed its sound psychometric properties across healthy adults, university students, and patients with insomnia, depression, or neurosis (28). Based on the Chinese population standard, a total PSQI score greater than 7 was used as the threshold for determining sleep disturbances. This threshold has been empirically shown to yield a recognition rate of 98.3% and an exclusion efficacy of 90.2% (29).

### 2.4 Statistical analysis

The study first characterized the scale dimension scores and socio-demographic indicators of the subject group using basic statistical analysis. Next, multidimensional network modeling techniques were employed to construct co-morbidity network models for anxiety, depression, and stress symptoms, integrating the dimensions of sleep disturbances to form a combined symptom network. This modeling approach effectively captures the dynamics of multidimensional associations within the psychopathological system, examining conditional associations that are challenging to adequately characterize using traditional regression models or latent variable frameworks (15). During the model construction phase, item redundancy tests were conducted using the networktools package (v1.5.1) for the R programming platform. Based on the discriminant criterion proposed, measurement redundancy was identified when the difference in Pearson correlation coefficients between two variables and other nodes met the 25% threshold. All data were standardized (mean = 0, SD = 1) prior to network estimation to ensure comparability across variables with different measurement scales. All data processing and modeling analyses were performed in the R 4.3.2 programming environment.

### 2.5 Network analysis

#### 2.5.1 Network estimation

In cross-sectional study designs, traditional methods often employ pairwise Markov random fields (PMRF) as an analytical framework. A Gaussian graphical model (GGM) was constructed to estimate the symptom network (12). In this model, nodes represent individual symptoms or PSQI components, and edges represent regularized partial correlations after conditioning on all other nodes. To enhance model fitting, the study applies the Extended Bayesian Information Criterion (EBIC) for optimal model selection, with the standardized adjustment parameter set to γ = 0.5, which represents a balance between model sparsity and accuracy in accordance with Epskamp et al.’s (2018) recommendation for psychological network analysis. Additionally, the LASSO regularization technique (30) is used to shrink small edges toward zero, thereby improving the sparsity and interpretability of the network structure. During the visualization process, the Fruchterman-Reingold spatial layout algorithm is employed to arrange the spatial distribution of nodes according to the gradient of correlation strength. The technical implementation relies on the R programming environment, utilizing the “graph” package (v1.9.8) and the “bootnet” package (v1.5.6) (31) to perform network parameter estimation and structural visualization. Bootstrap sampling was conducted with N = 1, 000 resamples to estimate edge stability and confidence intervals. In the final network map, nodes are represented geometrically, with edge widths corresponding to the strength of associations. Positive associations are depicted in blue, while negative associations are shown in red.

In network analysis, the quantification of node centrality is achieved through centrality metrics, which measure the degree of influence of a specific node’s connections within the network (32). Traditional centrality metrics typically include three categories: strength centrality, median centrality, and proximity centrality. However, some studies have indicated that the latter two may introduce biases when characterizing node influence (33). This study uses strength centrality as the primary evaluation dimension, emphasizing the strength of direct connections by summing the absolute connection weights between the target node and its adjacent nodes. The larger the value of this metric, the greater the node’s connectedness in the network. To further explore the interaction characteristics of cross-community nodes, this study utilizes the bridge function in the R package “networktools” (version 1.5.1) to assess bridge centrality (34). Bridge centrality defines bridge strength as the total connection strength between nodes within a community and those in other communities, highlighting the potential role of these nodes in connecting different network components. The degree to which node state changes are explained—i.e., the explained variance in predicting the variation of a target node based on the characteristics of its neighboring nodes—is ultimately quantified through the predictability computational model of the MGM toolkit (version 1.2-14) (35). In the network visualization, the area surrounding each node represents its predictability value.

#### 2.5.2 Network stability and accuracy

This study performs network stability and accuracy assessment using the R language Botnet package (version 1.5.6) and applies Bootstrap sampling techniques to systematically evaluate the robustness and estimation accuracy of the network structure. The analysis process consists of three core phases: First, case-dropping bootstrap was performed with N = 1, 000 resamples to assess edge connection strength and bridge node stability through a progressive data subset rejection strategy, ensuring that the overall network structure remains largely unaffected. The Correlation Stability Coefficient (CS-C) is then calculated as a quantitative measure of network interference resistance, which quantifies the proportion of cases that can be dropped while maintaining a correlation of 0.7 or higher with the original centrality index. According to the methodological standard, CS-C must be at least 0.25 to be considered acceptable for interpretation, and CS-C ≥ 0.5 is considered optimal. Second, the 95% confidence intervals (CIs) of edge weights were calculated using the nonparametric Bootstrap method (36), with the width of these intervals used to assess edge estimation precision. Narrow CIs (± 0.05) indicate high precision, while wider CIs suggest uncertainty in edge estimation. Third, Bootstrap pairwise comparisons were employed to analyze the statistical differences between the distribution of edge weights and centrality indexes, thereby examining the robustness of the results and identifying significant features of the network structure. Edge weights were interpreted with reference to their 95% CIs. Additionally, the Network Comparison Test (NCT) was conducted to compare global strength and network structure across subgroups, with p-values adjusted using the Bonferroni-Holm correction to control for Type I error. Finally, predictability values (R²) for all nodes were computed and reported as mean ± SD to indicate how well neighboring nodes predict each node’s variation.

#### 2.5.3 Network comparison

Finally, to investigate potential differences in network characteristics related to comorbid symptoms and disturbances among healthcare workers, we analyzed the data according to gender, years of practice, and educational background. Previous studies have shown that gender differences may influence emotion regulation through social role expectations (37), that years of practice are related to stages of adaptation to occupational stress (38), and that education level may be associated with cognitive resources and access to psychological interventions (39). All these factors may alter the connectivity patterns of symptom networks. A hierarchical comparison model was developed based on demographic characteristics: gender was categorized dichotomously (male/female), years in practice were divided into experience tiers with a ten-year cutoff (<10 years/≥10 years), and educational backgrounds were classified by degree level (Bachelor’s degree or lower/Master’s degree or higher). Using the Network Comparison Test Package (version 2.2.2) in R, a comparison network was constructed through 1, 000 iterations of Bootstrap resampling to quantitatively assess subgroup variability in terms of global strength (the sum of the absolute values of all edge weights) and network structure (the distribution pattern of edge weights) (40). The NCT provides permutation-based p-values for testing differences in global strength and individual edge weights between subgroups. Lastly, the Bonferroni-Holm correction algorithm was applied to control for Type I error inflation and assess differences in individual edge strength across the different networks.

## 3 Results

### 3.1 Characteristics of the study sample

**Table 1** presents the demographic characteristics of the participants in this study. Among the 1, 335 participants, the mean age was 34.04 years (SD = 6.36), with 323 (24.19%) identifying as male and 1, 012 (75.81%) as female. Regarding educational attainment, 1, 124 participants (84.19%) had a bachelor’s degree or lower, while 211 (15.81%) held a postgraduate degree or higher. In terms of mental health, 321 participants (24.04%) reported experiencing anxiety (Anxiety total score ≥ 8), 291 (21.80%) reported depression (Depression total score ≥ 8), 398 (29.81%) reported stress (Stress total score ≥ 8), and 1, 080 participants (80.90%) reported disturbances (PSQI total score ≥ 8). **Supplementary Table 1** presents the mean, standard deviation (SD), skewness, kurtosis, intensity, bridge intensity, and predictability of all items across the scales. No duplication of items was found within the co-morbidity networks for anxiety, depression, and stress, or in the combined network for disturbances.

**Table 1:** Demographic characteristics of the study participants (n = 1, 335).

Variables	Mean/N	SD/%
Age (year)	34.04	6.36
Anxiety	4.78	5.19
Depression	4.52	5.10
Stress	5.66	5.18
Pittsburgh Sleep Quality Index (PSQI)	11.36	4.05
Gender		
Male	323	24.19%
Female	1012	75.81%
BMI (kg/m2)		
Underweight (≦ 18.4)	252	18.88%
Normal weight (18.5–23.9)	883	66.14%
Overweight (≧ 24)	200	14.98%
Education level		
Undergraduate and below	1124	84.19%
Master’s degree and above	211	15.81%
Length of job (years)		
10 years or more	608	45.54%
Below 10 years	727	54.46%
Type of work		
Doctor	290	21.72%
Nurse	916	68.61%
Other medical technician	129	9.66%
Anxiety		
No anxiety (0–7)	1014	75.96%
With anxiety (8–21)	321	24.04%
Depression		
No depression (0–7)	1044	78.20%
With depression (8–21)	291	21.80%
Stress		
No stress (0–7)	937	70.19%
With stress (8–21)	398	29.81%
Pittsburgh sleep quality index (PSQI)		
Normal sleep (0–7)	255	19.10%
Have disturbances (8–21)	1080	80.90%

### 3.2 Stress, anxiety and depression network.

**Figure 1:**
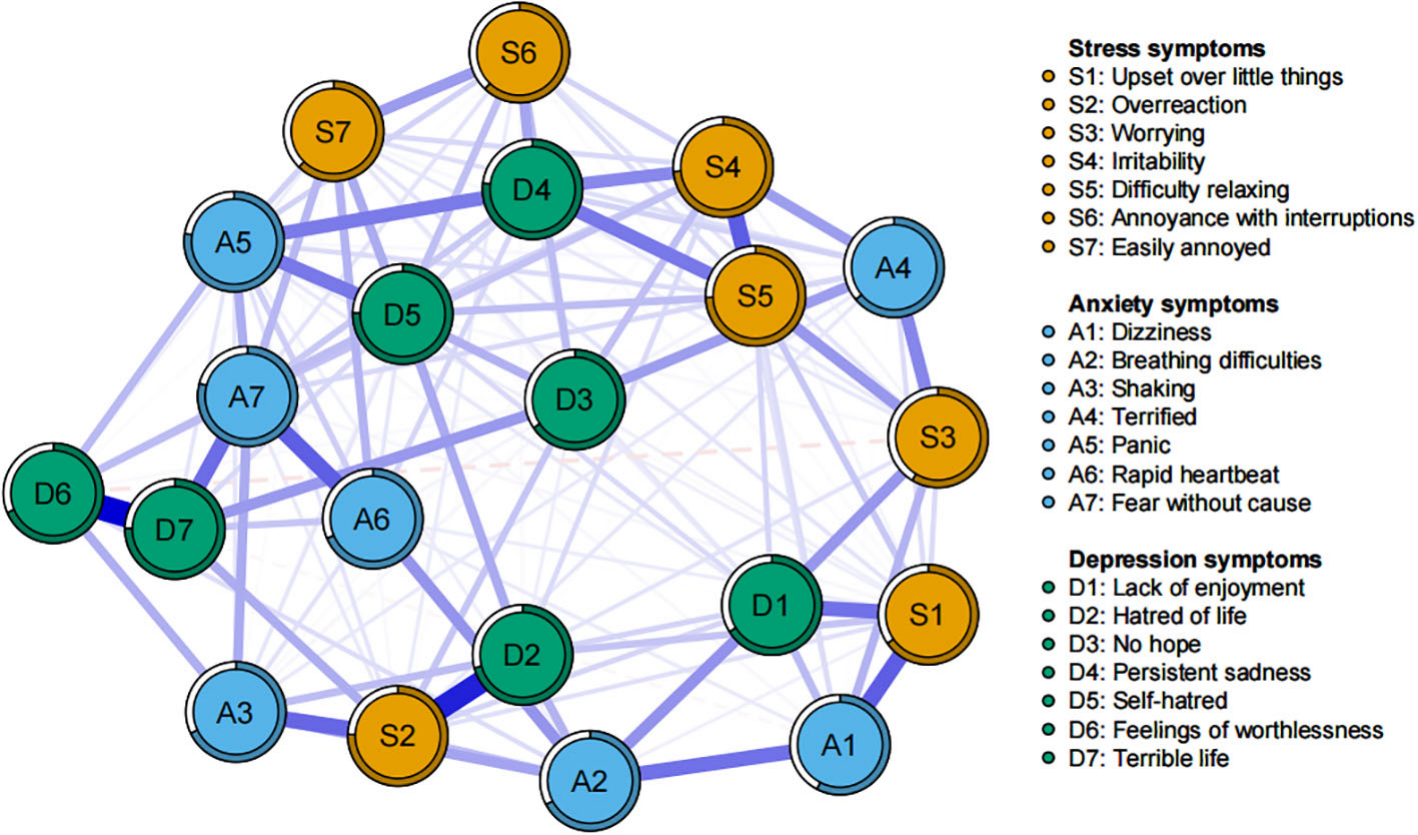
Network structure of stress, anxiety, and depression among study participants.

As shown in **Figure 1**, the network structure, which encompasses stress, anxiety, and depression-related questions, consists of 141 non-zero edges out of a possible 210 (network density = 0.67), with an average edge weight of 0.048. The network also reveals cross-category associations between stress, anxiety, and depression symptoms. For example, S1 (Upset over little things) is connected to A1 (Dizziness), indicating an association between anxiety-related physiological symptoms. Similarly, S3 (Worrying) is linked to A4 (Terrified), indicating that worry was associated with terror. S5 (Difficulty relaxing) is connected to D1 (Lack of enjoyment), reflecting the bidirectional relationship between stress and depression. Additionally, A2 (Breathing difficulties) is connected to D4 (Persistent sadness), suggesting overlap between anxiety and depression. These cross-category connections indicate the interconnectedness of these three constructs, suggesting that they are not entirely distinct but instead share common underlying processes (**Figure 1**).Furthermore, Fear without Cause (A7) and Panic (A5) exhibited the highest predictability values of 0.792 and 0.776, respectively, while Dizziness (A1) showed the lowest predictability at 0.578. The average predictability was 0.692, indicating that, on average, more than half of the variance in the nodes was explained by their neighboring nodes (**Supplementary Table 2**). The two most prominent edges within specific neighborhoods were Feelings of worthlessness - Terrible life (D6-D7) and Overreaction - Hatred of life (S2-D2) (**Supplementary Figure 2**).As shown in **Supplementary Figure 6**, symptoms of stress, anxiety, and depression exhibit significant node strength and bridge strength, indicating their high connectedness in the network. The stability analysis indicates that these metrics remain robust even as the sample size decreases. Additionally, the confidence intervals for the edges further substantiate the significance of the connections between these symptoms.

**Figure 2:**
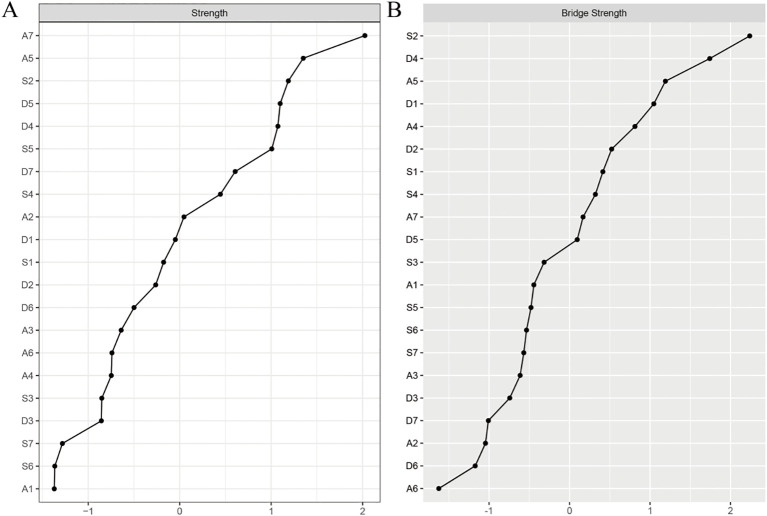
Standardized strength and bridge strength centrality of network structure of stress, anxiety, and depression among study participants (z-scores). **(A)** strength centrality; **(B)** bridge strength centrality.

The centrality graph (**Figure 2A**) indicated that Fear without cause (A7) exhibited the highest centrality, followed by Panic (A5) and Overreaction (S2), suggesting that these nodes show the highest connectedness within the network of comorbid symptoms. According to Jones (50), bridge symptoms were selected using the 80th percentile threshold for bridge centrality. As shown in **Figure 2B**, Overreaction (S2), Persistent sadness (D4), Panic (A5), and Lack of enjoyment (D1) exhibited the highest bridge strengths, identifying them as key bridging symptoms within the network of comorbidities. Bootstrap difference tests for node strength and bridge strength (**Supplementary Figure 6**) further confirmed that these nodes were statistically significantly stronger than other nodes in the network.

### 3.3 Network of stress, anxiety, depression, and disturbances

**Figure 3:**
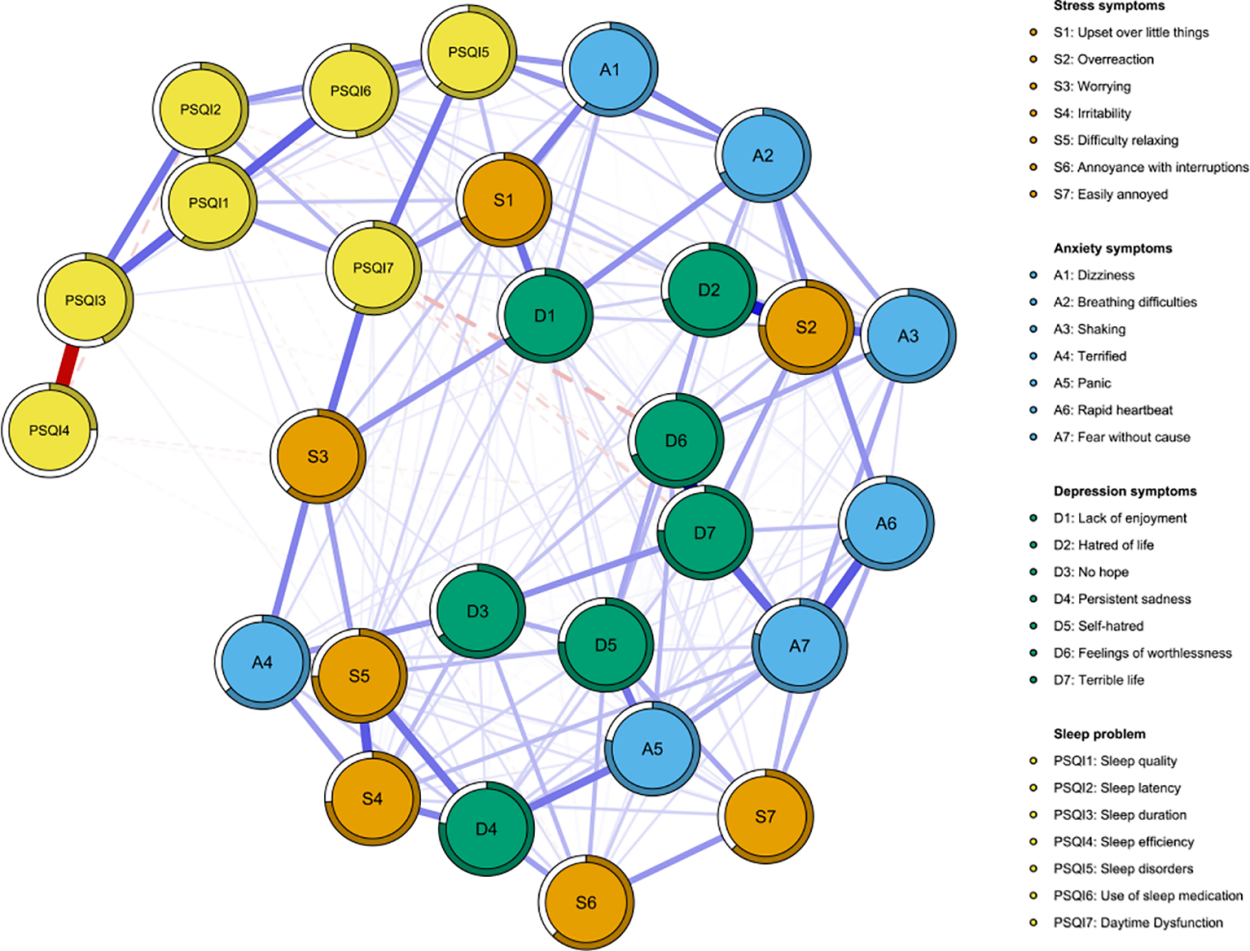
Network structure of comorbid symptoms and disturbance among study participants.

The network of comorbid symptoms and disturbances is illustrated in **Figure 3**, with stability and accuracy tests provided in **Figure 3**. Regarding power centralization, Fear without cause (A7), Terrible life (D7), and Panic (A5) emerged as the most central comorbid symptoms. Meanwhile, Daytime Dysfunction (PSQI7) and Sleep quality (PSQI1) were identified as the two central components in the network (**Supplementary Figure 3**).Additionally, the three most prominent edges were observed within specific neighborhoods: Feelings of worthlessness - Terrible life (D6-D7), Overreaction - Hatred of life (S2-D2), and Sleep quality - Sleep latency (PSQI1-PSQI2) (**Supplementary Figure 4**).

In terms of conditional associations, Daytime Dysfunction (PSQI7) and Sleep disturbances (PSQI5) exhibited the strongest associations with comorbid symptoms. Additionally, an assessment of the individual associations between each specific PSQI node and the broader community of comorbid symptoms revealed that Sleep disturbances (PSQI5) had the strongest bridging power, connecting the sleep and psychological symptom communities (**Supplementary Table 3**; **Supplementary Figure 5**). Daytime Dysfunction (PSQI7) and Sleep disturbances (PSQI5) were predominantly positively correlated with comorbid symptoms. Consequently, Daytime Dysfunction (PSQI7) and Sleep disturbances (PSQI5) were identified as central sleep-related nodes associated with psychological symptoms.

### 3.4 Comparison of mental health status based on gender, level of education and length of employment (years)

NCT revealed invariance in overall strength and network structure across the three groups (**Supplementary Figures 8**-**10**). In terms of overall strength comparisons between the subsamples, no significant differences were observed in global network strength (males: 13.73 vs females: 13.84, p = 0.818; undergraduate and below: 13.85 vs master’s degree and above: 13.63, p = 0.751; <10 years: 13.86 vs ≥10 years: 14.30, p = 0.207).Regarding the comparison of network structure, no significant differences were found between the three subsamples for gender, length of employment and education level (gender: M = 0.19, p = 0.268; education level: M = 0.25, p = 0.056; length of employment: M = 0.19, p = 0.110). Furthermore, after applying the Bonferroni-Holm correction, all edge weights in the gender and length of employment subsamples remained non-significant (p > 0.05).

## 4 Discussion

In this study, we explored the relationship between co-morbid symptoms and disturbances through a network analysis approach. The results showed that more than one-fifth of the participants experienced depression, anxiety, and stress, while over 80% reported disturbances. Specifically, the prevalence rates of depression, anxiety, stress, and disturbances were 21.80%, 24.04%, 29.81%, and 80.90%, respectively. Consistent with similar previous studies, this study assessed the core and bridging symptoms within the combined network of depression, anxiety, and stress (25, 41), identifying central and bridge nodes associated with psychological symptoms. Two distinct communities were identified in this study: one comprising symptoms of anxiety and stress, and the other encompassing depression. The DASS-21 scale assessed seven aspects of disturbances: subjective sleep quality, sleep latency, sleep duration, sleep efficiency, disturbances, sleep medication use, and daytime dysfunction. Strong connections were found within specific communities of psychological symptoms, consistent with previous studies addressing network structures (42, 43).

### 4.1 Anxiety, depression and stress network results

The results of the network analysis revealed a significant dominance of causeless fear (A7) in centrality strength, indicating its high connectedness within the co-morbid network of anxiety, depression, and stress. This symptom appears to be more prevalent among healthcare workers, possibly because their daily duties involve multiple stressors—including high workloads, the need to manage emergencies, and complex doctor–patient relationships. Moreover, the healthcare environment itself is characterized by considerable uncertainty and potential threats, factors that may increase susceptibility to “causeless fear” (44). For instance, healthcare workers often encounter uncertainties in patient conditions, sudden medical emergencies, and potential conflicts between doctors and patients. All these factors may be associated with a state of constant alertness, even without any clear danger, making them prone to developing unfounded fears. This unique ‘continuous exposure to uncertainty’ in the healthcare profession might be a possible explanation for ‘unjustified fear’ showing high centrality. Second, existing studies have demonstrated a close association between generalized anxiety disorder and the emergence of fear-related emotions. (43). Extensive research has demonstrated that the amygdala is significantly involved in fear-related response circuits (45, 46) and that unwarranted fear may lead to overactivation of the amygdala, which, in turn, exacerbates anxiety symptoms (47). A Canadian experimental study showed that stress can contribute to generalized fear (48). Network analysis by Yang Y et al. revealed that the feeling of fear had high centrality within the depression-anxiety network (49). This finding differs somewhat from our results, possibly due to the use of different assessment scales for the symptoms. Moreover, the inclusion of stress-related factors in our study’s network analysis further suggests the relevance of “causeless fear” to depression, anxiety, and stress symptoms, offering a more comprehensive understanding of its role in mental health-related domains. Additionally, Panic (A5) and Overreaction (S2) emerged as the second most important core symptoms, both showing high bridge strengths and functioning as significant bridging symptoms. Panic is a hallmark of anxiety disorders, characterized by panic attacks (50). For healthcare workers, the stressful and fast-paced working environment, coupled with the responsibility of dealing with patient life and death, may make them more prone to experiencing intense physical and psychological reactions in emergency situations, which may be associated with panic. Network analysis by Cha EJ et al. identified a strong association between depression and panic (50). Furthermore, chronic stress has been associated with prolonged symptom duration and poor treatment response in panic disorder, potentially leading to co-morbid anxiety or depressive symptoms (51). This further substantiates the association between panic and the co-morbidity of anxiety-depressive stress. Chronic anxiety patients are often subjectively and physiologically overreactive to stimuli (52). One predictor of depression is rejection sensitivity, which manifests as an overreaction to potential rejection situations (53). This suggests that overreaction is deeply linked to depressive symptoms, supporting our findings. When healthcare workers encounter setbacks, criticism or negative feedback from patients and their families during their work, due to the prolonged state of mental tension, they may be more prone to having “excessive reactions” in their emotions. Such reactions not only affect their own emotions but may also further intensify work pressure and interpersonal conflicts. Moreover, Sun C et al.’s network analysis confirmed that overreaction is one of the bridging symptoms for the co-morbid symptoms of depression, anxiety, and stress (54), which is consistent with our results.

Nodal bridging intensity centrality may offer valuable insights into identifying key bridging symptoms in the connectivity and cross-domain associations among symptoms. In addition to panic and overreactivity, several other bridging symptoms have emerged in the current network, including persistent sadness and lack of enjoyment. Persistent sadness and loss of interest are key symptomatic manifestations of depression (55, 56), and persistent sadness is also a prominent feature within the anxiety disorder community (57). Among the healthcare workers, prolonged shift work, high workloads, and repeated exposure to illness and death can all lead to emotional exhaustion, which in turn may cause “ongoing sadness” and “lack of joy”. These symptoms serve as a bridge, potentially connecting the sense of job burnout with deeper depression and anxiety. For instance, healthcare workers who remain in this state for a long time may lose interest in the work they once loved, and even become numb to other aspects of life, thereby exacerbating their psychological distress. Previous studies have shown that persistent sadness and anhedonia can intensify the interplay among anxiety, depression, and stress by disrupting emotion regulation, cognitive functioning, and behavioral responses (58). A cross-sectional study has shown that grief regulation plays a significant role in anxiety and depressive symptoms (59), indicating that individuals who struggle with regulating sadness are more likely to experience symptoms of both depression and anxiety. This finding is consistent with our findings. It is worth noting that existing network analysis studies have explored the comorbidity mechanisms of the aforementioned issues among healthcare workers. For instance, using the PHQ-9 and GAD-7 scales, Li et al. analyzed the associations between stress, burnout, anxiety, and depression among Chinese resident physicians (60). Pu et al. focused on the network characteristics of anxiety and depression in nurses during the COVID-19 pandemic relief period (61). Other studies have validated the interactions between core symptoms of physician psychological distress, such as “helplessness, “ and stress, burnout, depression, and anxiety (62).

Our findings align with these studies regarding the strong connectivity between depressive and anxious nodes, yet several differences emerge due to variations in sample composition and occupational settings. While Li et al. (60) and Pu et al. (61) identified “fatigue” or “nervousness” as central symptoms during high-intensity clinical or pandemic periods, our study—conducted in a normalized healthcare environment—reveals a more complex symptom interaction. These discrepancies may stem from the different measurement tools (e.g., DASS-21 vs. PHQ/GAD) and the specific time periods of data collection. Notably, these prior studies either focus on specific subgroups or fail to delve into the unique symptom interaction patterns inherent in the healthcare occupational environment —— such as the causeless fear” identified in our study. This symptom is closely related to the persistent uncertainty in the occupational context and has not been highlighted in prior research. By clarifying the core and bridging symptoms related to the occupation in this group, our study provides more targeted empirical evidence for understanding the interaction mechanisms of anxiety, depression, and stress among healthcare workers.

### 4.2 Anxiety, depression, stress, and disturbances network results

According to the World Health Organization (WHO), approximately 30% to 35% of the global population reports experiencing disturbances. Healthcare workers, in particular, exhibit a high prevalence of sleep disturbances. A systematic review and meta-analysis revealed that the global prevalence of insomnia among healthcare workers may reach 33% (63), underscoring the significance of our study. Our findings indicate that daytime dysfunction and disturbances represent central sleep-related nodes associated with psychological symptoms within the network. This highlights their role as an intersection point between mental health and sleep-related issues. Furthermore, these two factors emerged as prominent bridge nodes when quantifying the relationship between disturbances and symptoms of anxiety, depression, and stress. Daytime dysfunction, a common clinical manifestation of disturbances (64), has been reported to be associated with the structure of the depression-sleep comorbidity network. Its node strength is crucial in facilitating the interactions between symptoms across diagnoses in the cross-diagnostic symptom interaction model (65). Research has shown that higher levels of stress are associated with increased sleep disturbances and daytime dysfunction (66). Similarly, a study examining anxiety, depression, and disturbances among college students yielded consistent findings (29). These results suggest a moderate link between daytime dysfunction resulting from sleep disturbances and mental health issues, providing strong support for the present study. Sleep disturbance is a key symptom in patients with depression, and a bidirectional correlation between the two has been well-documented (67, 68). Additionally, a study involving healthcare professionals demonstrated a significant association between depressive symptoms and sleep disturbances (69). Another study found that individuals with anxiety often experience concurrent disturbances (70). Furthermore, a neuroscience study by Sanford LD et al. highlighted a strong association between stress and sleep disturbances. Collectively, these findings suggest a significant connection between disturbances—an important manifestation of disturbances—and psychological issues. Sleep hygiene improvements, such as regular sleep–wake schedules, caffeine avoidance, and reduced nocturnal light exposure, effectively enhance sleep quality, thereby regulating emotional, cognitive, and behavioral responses and disrupting the complex symptom network. Previous studies have confirmed that sleep hygiene interventions not only relieve insomnia but also significantly ameliorate symptoms of anxiety, depression, and stress, highlighting their clinical value (71). However, to the best of our knowledge, no studies have employed network analysis to link disturbances with these co-occurring symptoms. This gap in the literature suggests that our study may provide a valuable contribution to existing research.

NCT analyses and centrality quantification suggested that daytime dysfunction and sleep disturbance were important sleep-related correlates of psychological symptoms within our study population. These findings suggest their potential importance in similar contexts, though further research is needed to confirm their broad applicability. Furthermore, when comparing the intensity of behaviors and their impact on mental health symptoms across subgroups, no significant differences in disturbances were found between the subsamples for gender and length of job. In response to this discrepancy, we speculate that it may be associated with the following factors: First, healthcare workers with higher education levels may differ in psychological resilience and cognitive capacity, which may attenuate the strength of the association between sleep disturbances and anxiety. Second, individuals with higher educational attainment may face different types of occupational pressure, whereas those with lower education levels are more focused on high-intensity clinical work. The differences in types of stress may reshape the patterns of symptom interaction. Third, those with higher education levels may have different access to psychological resources, which may disrupt the negative cycles between symptoms. However, differences were observed in the subsample based on education level. Previous studies have indicated that gender plays a significant moderating role in the relationship between disturbances and co-occurring symptoms (72). A study by Wei Li et al. found that the proportion of anxiety and depressive symptoms was significantly higher among individuals with a shorter length of job compared to those with a longer duration of employment, who reported fewer disturbances (73). These findings differ somewhat from ours, possibly due to our innovative use of network analysis to examine associations between symptoms, which represents a novel methodological approach. Further research is needed to explore these relationships in greater depth.

### 4.3 Strengths, limitations and prospects

Our study has several strengths. First, it employs psychometric network modeling to successfully identify the central symptoms of three typical mental disorders, as well as their bridging symptoms, thereby constructing a dynamic map of associations among these symptoms. Second, the incorporation of PSQI components into the network extends previous research by examining sleep disturbances alongside psychological symptoms rather than treating sleep only as a total score or peripheral outcome. These findings may help generate hypotheses for future intervention studies. For instance, exposure therapy within Cognitive Behavioral Therapy (CBT) is an effective intervention for core symptoms such as “causeless fear.” Studies have shown that CBT is highly effective in managing occupational stress, particularly cognitive-behavioral interventions that can significantly alleviate psychological stress and anxiety reactions in healthcare workers (74). For bridging symptoms like “overreaction” and “persistent sadness, “ emotion regulation training can be employed to block the cross-dimensional transmission of symptoms, a strategy that aligns with the emotion regulation techniques emphasized in CBT. For sleep-related issues such as “daytime dysfunction, “ sleep hygiene interventions, including regular sleep schedules and reducing exposure to blue light at night, can be implemented to improve sleep quality, thereby alleviating psychological symptoms in healthcare workers (71). Additionally, a comparative web-based test was conducted to further investigate the differential impact and generalizability of these behaviors across a population of healthcare professionals. It is worth noting that this study expands and innovates on previous network research. First, unlike studies focusing solely on anxiety and depression in healthcare workers (60, 61, 75), we integrated stress and sleep dimensions to systematically reveal their interactive mechanisms in the occupational context, filling gaps in multidimensional comorbidity network analysis. Second, while general population studies (76) identified “sad mood” and “excessive worry” as central symptoms, our study highlights “causeless fear” as core—reflecting medical settings’ unique uncertainty and high risks, which amplify such fear’s central role, offering new insights into healthcare workers’ psychopathology. Third, though “overreaction” was previously recognized as a bridging node for anxiety, depression, and stress (77), we further examined its links with sleep disturbances in this group, clarifying how occupational stress exacerbates cross-dimensional transmission and refining the theoretical framework. These findings represent an incremental contribution: constructing the first comprehensive network model for healthcare workers, integrating fragmented mental health and sleep research to inform precise interventions.

However, our study has some limitations. Methodologically, the cross-sectional design, while effective in capturing patterns of Bootnet bootstrap symptom co-occurrence, is constrained by the absence of time-series data. This limits the ability to establish causal relationships between variables. We cannot definitively determine whether the core symptoms identified in this study act as drivers of other symptoms. This inability to make causal inferences may limit a deeper understanding of the mechanisms underlying comorbidity. Future cohort studies are needed to reveal the causal pathways between variables. Theoretically, the current model predominantly focuses on risk factors; in future studies, it would be beneficial to integrate protective factors and develop a dual-path risk-protection model to enhance explanatory power. Additionally, there are still some potential confounding factors in this study that have not been fully considered. For example, factors such as shift work, job satisfaction, work intensity, and occupational exposure risk may all have complex associations with anxiety, depression, stress, and sleep disturbances, thereby affecting the results we observed. Regarding the issue of sample representativeness, although a multi-stage sampling method was employed to control for selection bias, the research sample was limited to medical institutions in Fujian Province, and the sample included a high proportion of female participants and nurses. Therefore, the findings should be generalized cautiously to healthcare workers in other regions, cultural backgrounds, or medical systems. The prevalence, interrelationships, and influencing factors of mental health symptoms may vary significantly in different geographical locations and medical environments. These research results may not be directly generalized to healthcare professionals in other provinces or countries. Therefore, when applying these results to a broader group of medical professionals, caution should be exercised. Future research can adopt a multi-center sampling method, covering different economic levels in the eastern, central, and western regions, to verify the general applicability of the research results. Additionally, the use of the “Questionnaire Star” online platform for questionnaire distribution may introduce selection bias, potentially overrepresenting healthcare workers who are familiar with online operations and highly motivated to participate, while underrepresenting those who are less familiar with online platforms or have lower participation willingness. In terms of data quality, this study employed self-reported questionnaires to collect information, which are prone to potential response bias. On one hand, healthcare workers may experience social desirability bias due to societal expectations associated with their professional roles, potentially leading participants to underreport symptom severity. On the other hand, the inherent limitations of subjective assessments may compromise the accuracy of the data. It is recommended that future research incorporate objective physiological indicators for multimodal validation. Regarding the subgroup analyses, future research may further explore the interactions between education level, years of work, education level and other factors. Additionally, longitudinal studies could track the dynamic changes in symptom networks across different subgroups to clarify the impact of other factors on symptom evolution trajectories. This would provide a more precise basis for developing stratified and dynamic intervention strategies. By modeling the symptom networks of anxiety, depression, and stress in healthcare workers, this study not only elucidates the interaction patterns of co-morbid symptoms and disturbances but also proposes an innovative dynamic regulation model of symptom clusters. This theoretical framework challenges the traditional single-symptom research paradigm and provides an evidence-based foundation for the development of targeted mental health intervention strategies. Specifically, it offers significant application value in constructing psychological crisis early-warning systems for high-stress occupational groups.

Based on our network findings, we propose the following targeted interventions to mitigate the comorbidity of psychological distress among healthcare workers. First, given that “causeless fear” and “panic” are identified as core symptoms, priority should be given to symptom-focused psychological first aid. Specifically, evidence-based interventions such as exposure therapy or CBT can be implemented to help staff deconstruct irrational fears rooted in occupational uncertainty (60, 62). Second, to address bridging symptoms like “overreaction, “ hospital-based mindfulness training or emotion regulation programs are recommended to block the symptomatic “spillover” from stress to depression (58, 61). Finally, addressing “daytime dysfunction” requires workplace-level structural actions. Rather than general advice, hospital administrations should implement systematic sleep hygiene education and optimize shift-scheduling systems to ensure mandatory recovery periods (61). This stepped-care logic—ranging from individualized psychological support for core symptoms to organizational-level policy adjustments—provides a more concrete and implementable framework for improving the mental resilience of the healthcare workforce.

## 5 Conclusion

Key symptoms, such as causeless fear and panic, were central within the psychological symptom network, while symptoms such as persistent sadness and lack of enjoyment showed relatively high bridge strength. Incorporating PSQI components into the network model revealed associations between sleep-related nodes and psychological symptoms, particularly daytime dysfunction and sleep disturbances. These findings should be interpreted as cross-sectional symptom-level associations rather than causal relationships. Future longitudinal and intervention studies are needed to examine whether these symptoms play a role in the development or maintenance of psychological distress and disturbances among healthcare workers.

## Data Availability

The original contributions presented in the study are included in the article/**Supplementary Material**. Further inquiries can be directed to the corresponding authors.
