# Supplementary material for: Network analysis of anxiety, depression, stress, and sleep disturbances in healthcare workers
Source: Front Psychiatry. 2026 Jun 4;17:1726751. doi: 10.3389/fpsyt.2026.1726751 (PMC13275697; doi:10.3389/fpsyt.2026.1726751)
Supplement: Supplementary file 1 [file SupplementaryFile1.docx]

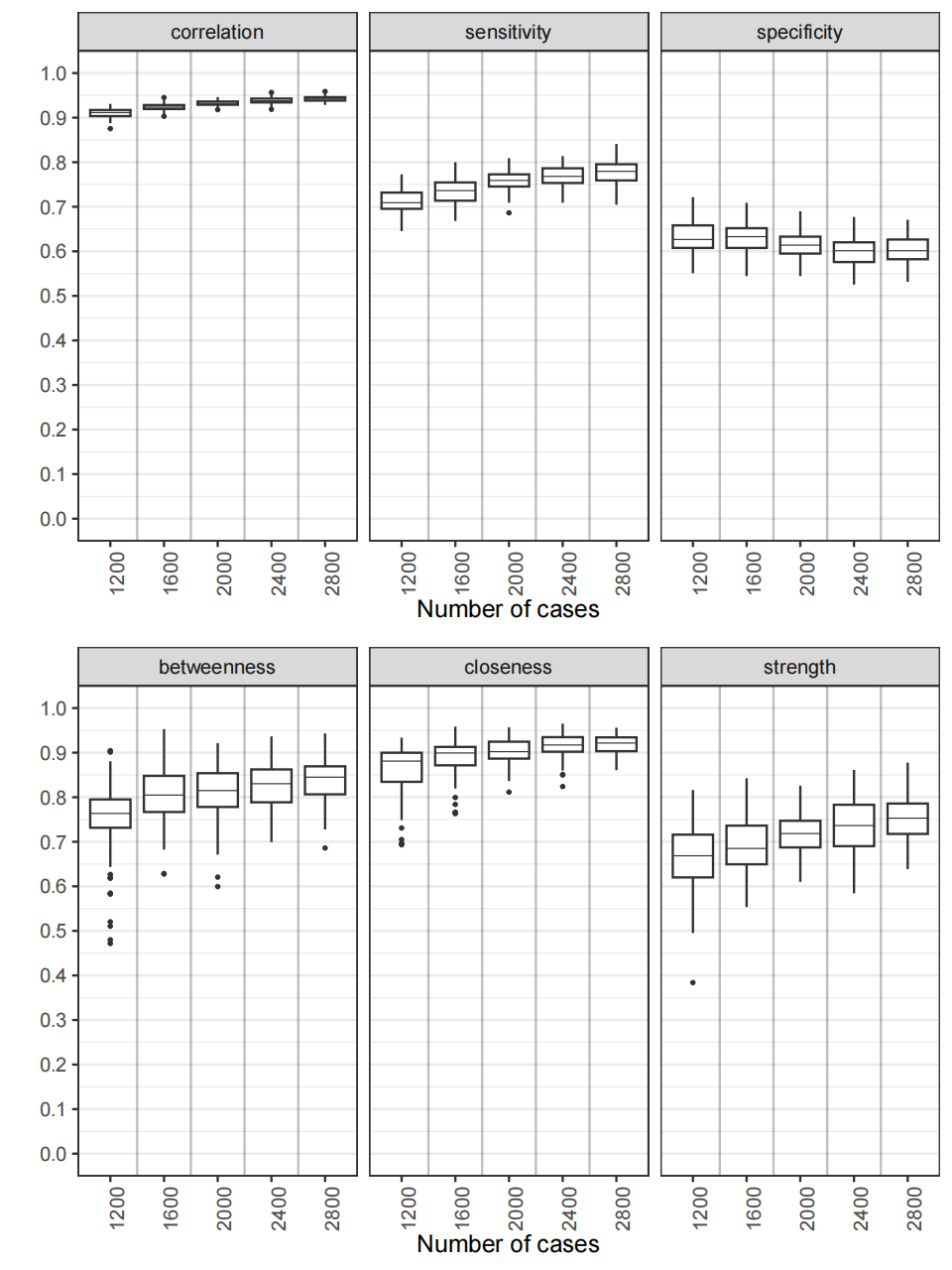


**Figure S1.** The power analysis simulation results of Comorbid symptoms and sleep problem network.


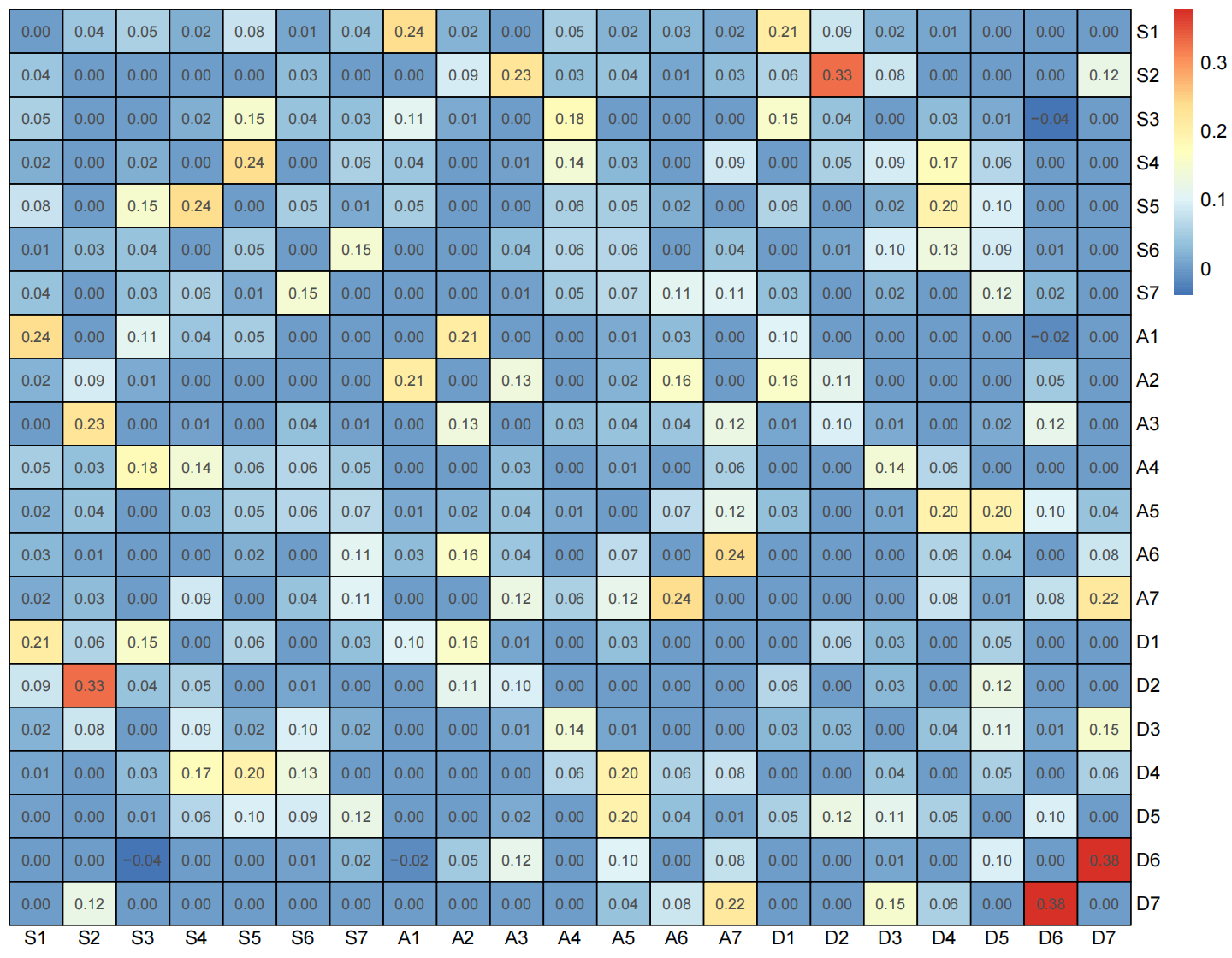


**Figure S2.** Estimated edge weights of stress, anxiety, and depression among study participants.


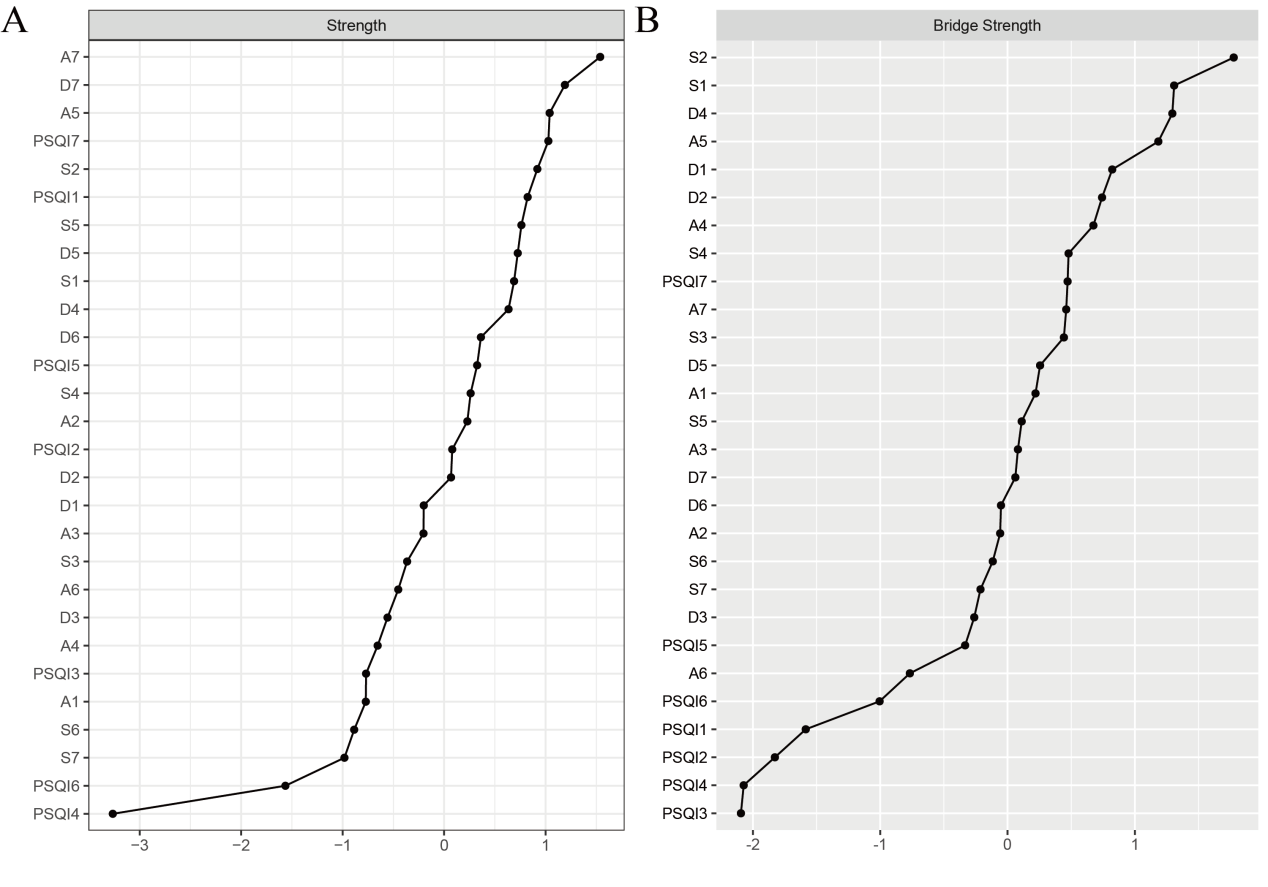


**Figure S3.** Standardized strength and bridge strength centrality of network structure of Comorbid symptoms and sleep problem network among study participants (z-scores). (A) strength centrality; (B) bridge strength centrality.


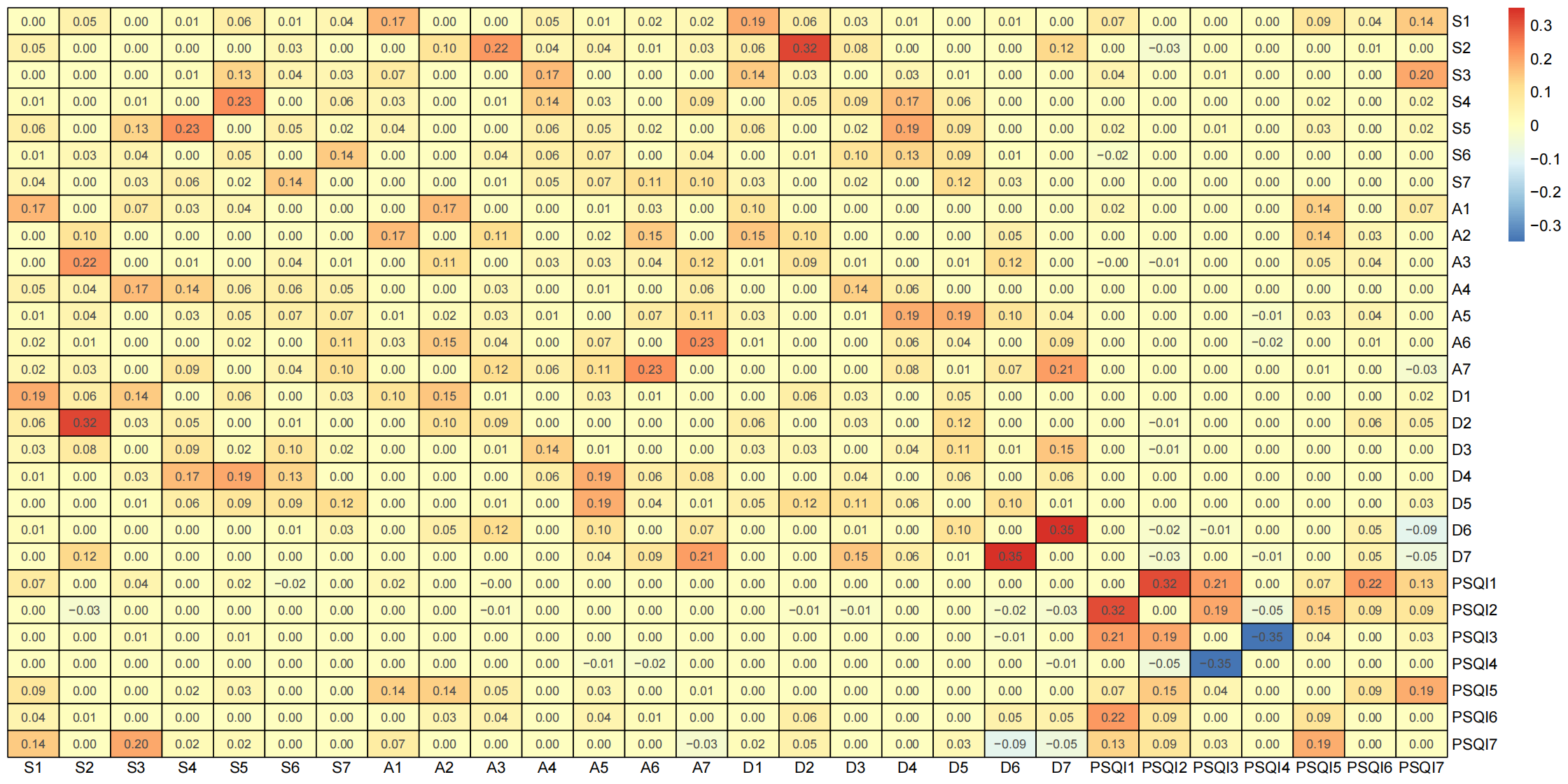


**Figure S4.** Estimated edge weights of comorbid symptoms and sleep problem among study participants.


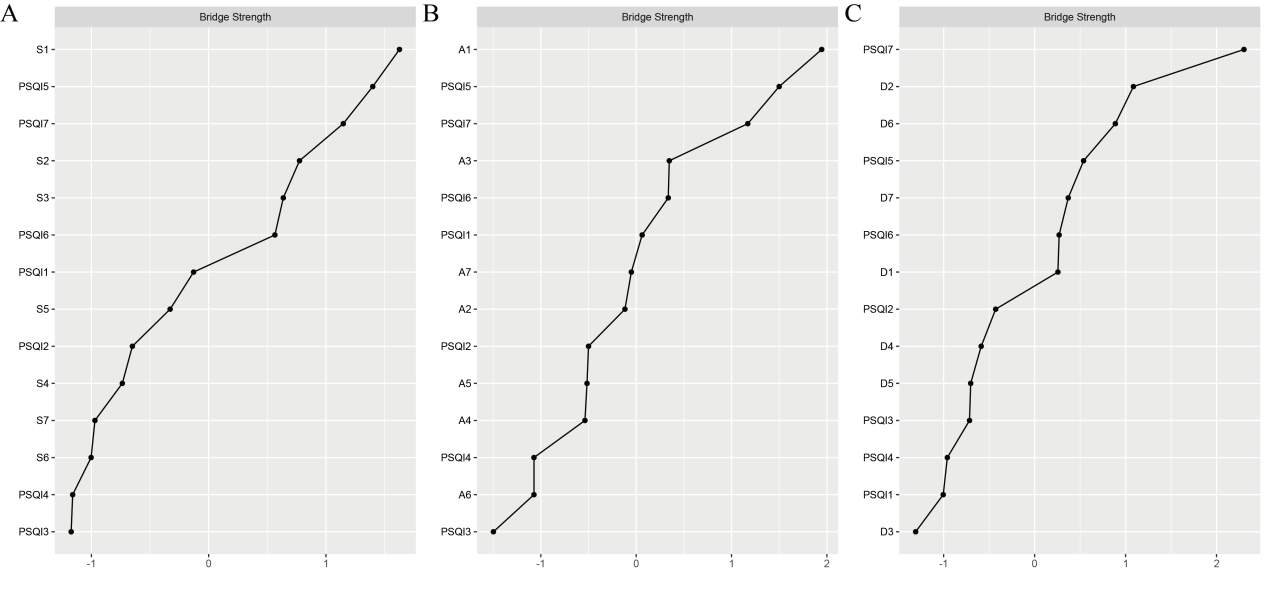
**Figure S5.** Standardized bridge strength centrality plot between sleep problem to Symptoms of Anxiety, Depression, Sleep problems (z-scores).

**
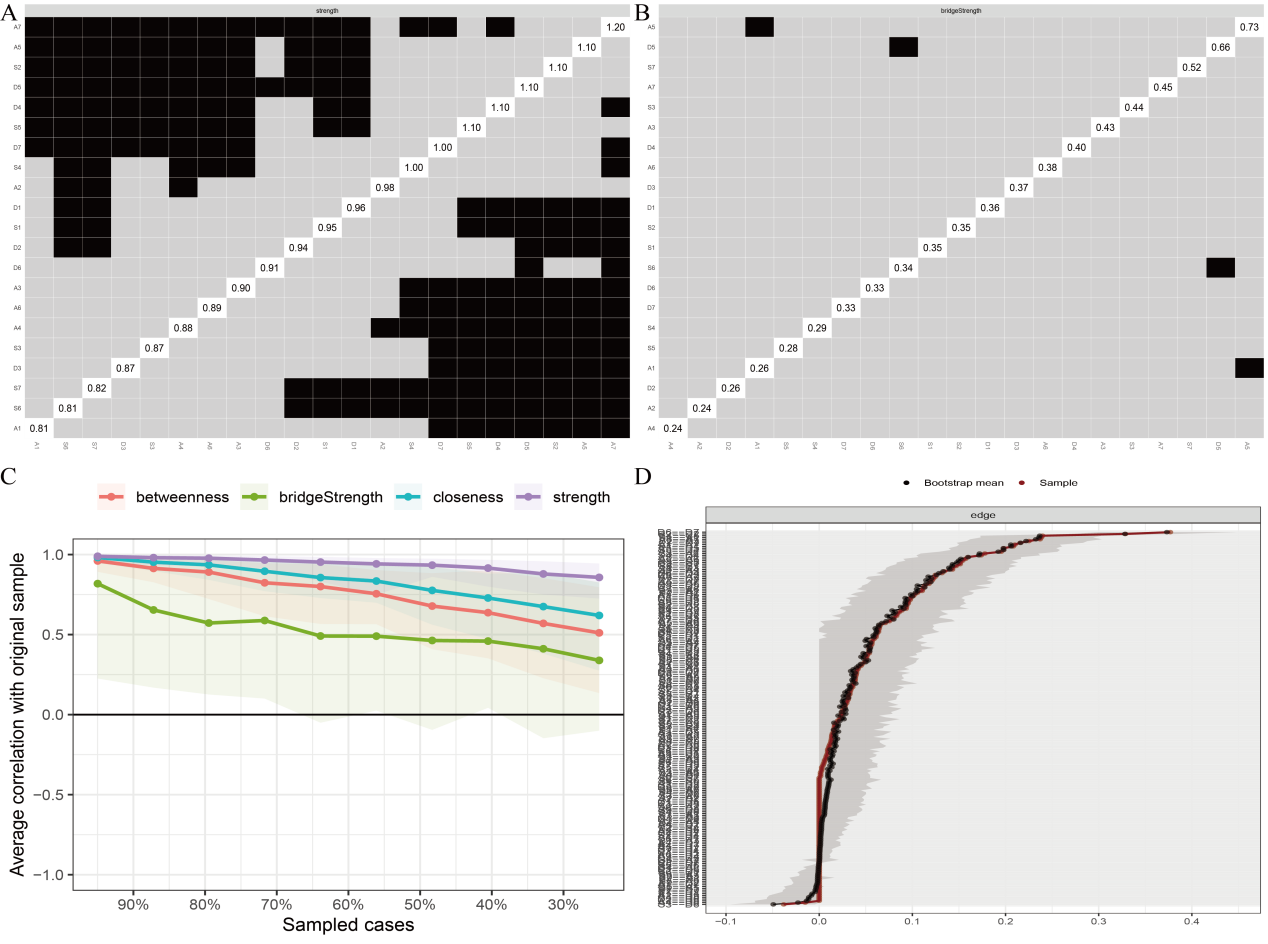
Figure S6.** Results of Network Stability and Accuracy for stress, anxiety, and depression. (A) Nonparametric bootstrapped difference test for strength. (B) Nonparametric bootstrapped difference test for bridge strength. Gray boxes indicate that node strength/bridge strength does not show significant differences, while black boxes indicate significant differences. The numbers within the white boxes (i.e., along the diagonal) represent the values of node strength/bridge strength. (C) Post-hoc analysis of the stability of node strength and bridge strength. The x-axis shows the percentage of the original sample used at each step, while the y-axis reflects the average correlations between the centrality metrics in the original network and those from the re-estimated networks after excluding progressively larger percentages of cases. The lines represent the correlations for strength and bridge strength. (D) Bootstrapped 95% confidence intervals for estimated edges. The red line indicates the edge estimated from the sample, and the gray area represents the 95% bootstrapped confidence interval. The x-axis shows the edges, with specific edges identified along the y-axis by the gray lines.


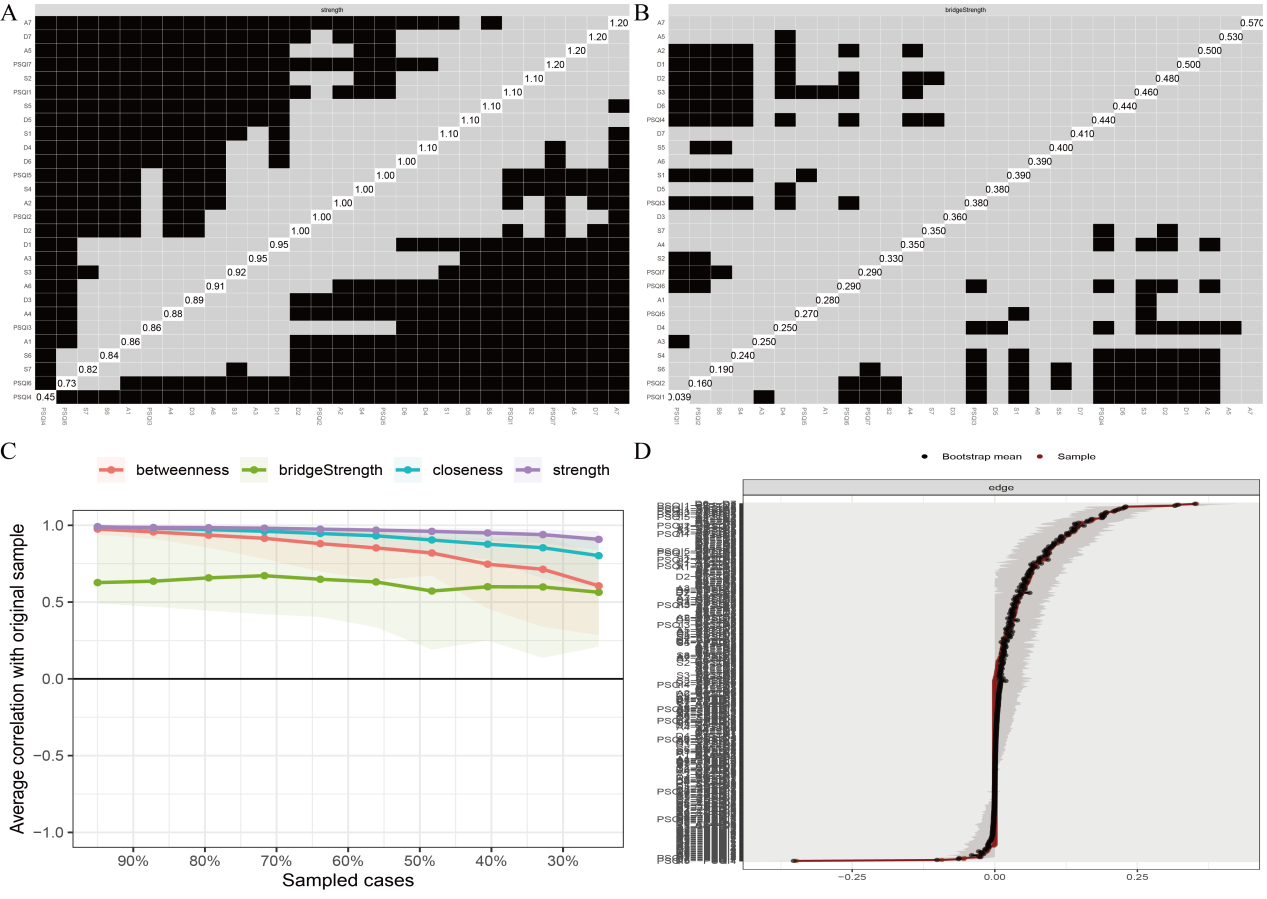


**Figure S7.** Results of Network Stability and Accuracy for Comorbid symptoms and sleep problem. (A) Nonparametric bootstrapped difference test for strength. (B) Nonparametric bootstrapped difference test for bridge strength. Gray boxes indicate that node strength/bridge strength does not show significant differences, while black boxes indicate significant differences. The numbers within the white boxes (i.e., along the diagonal) represent the values of node strength/bridge strength. (C) Post-hoc analysis of the stability of node strength and bridge strength. The x-axis shows the percentage of the original sample used at each step, while the y-axis reflects the average correlations between the centrality metrics in the original network and those from the re-estimated networks after excluding progressively larger percentages of cases. The lines represent the correlations for strength and bridge strength. (D) Bootstrapped 95% confidence intervals for estimated edges. The red line indicates the edge estimated from the sample, and the gray area represents the 95% bootstrapped confidence interval. The x-axis shows the edges, with specific edges identified along the y-axis by the gray lines.


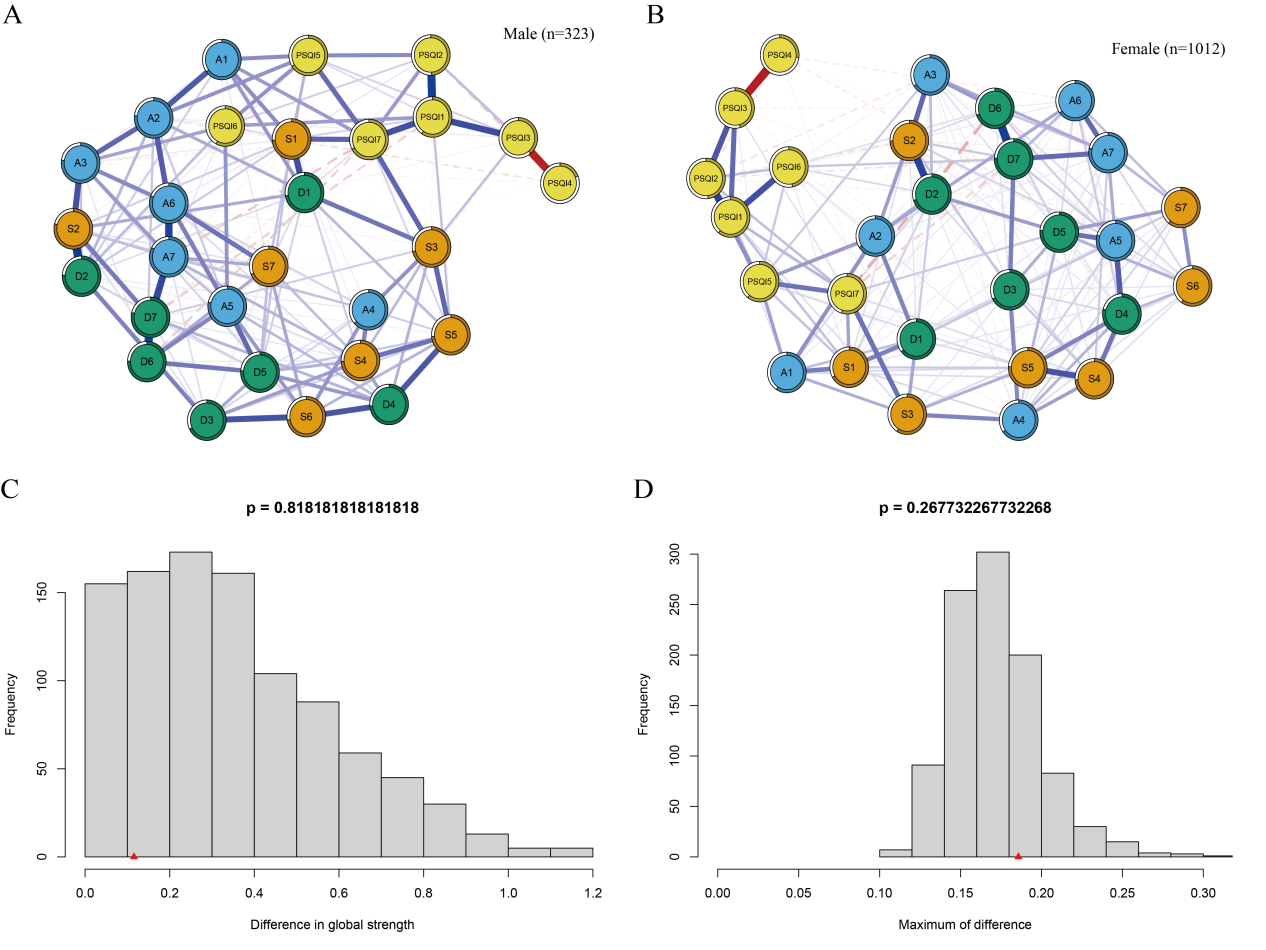


**Figure S8.** Network structure comparison based on gender. (A) Estimated network model for males (n = 323). (B) Estimated network model for females (n = 1012). (C) Bootstrap plot illustrating the difference in global strength, which was not statistically significant (global strength for males: 13.73; for females: 13.84; p = 0.818). (D) Bootstrap plot of the difference in network structure, showing no significant difference (M = 0.19, p = 0.268). The invariance of edge weights was assessed using permutation tests, yielding p values for each edge comparison. All Bonferroni-Holm adjusted p values were more than 0.05.


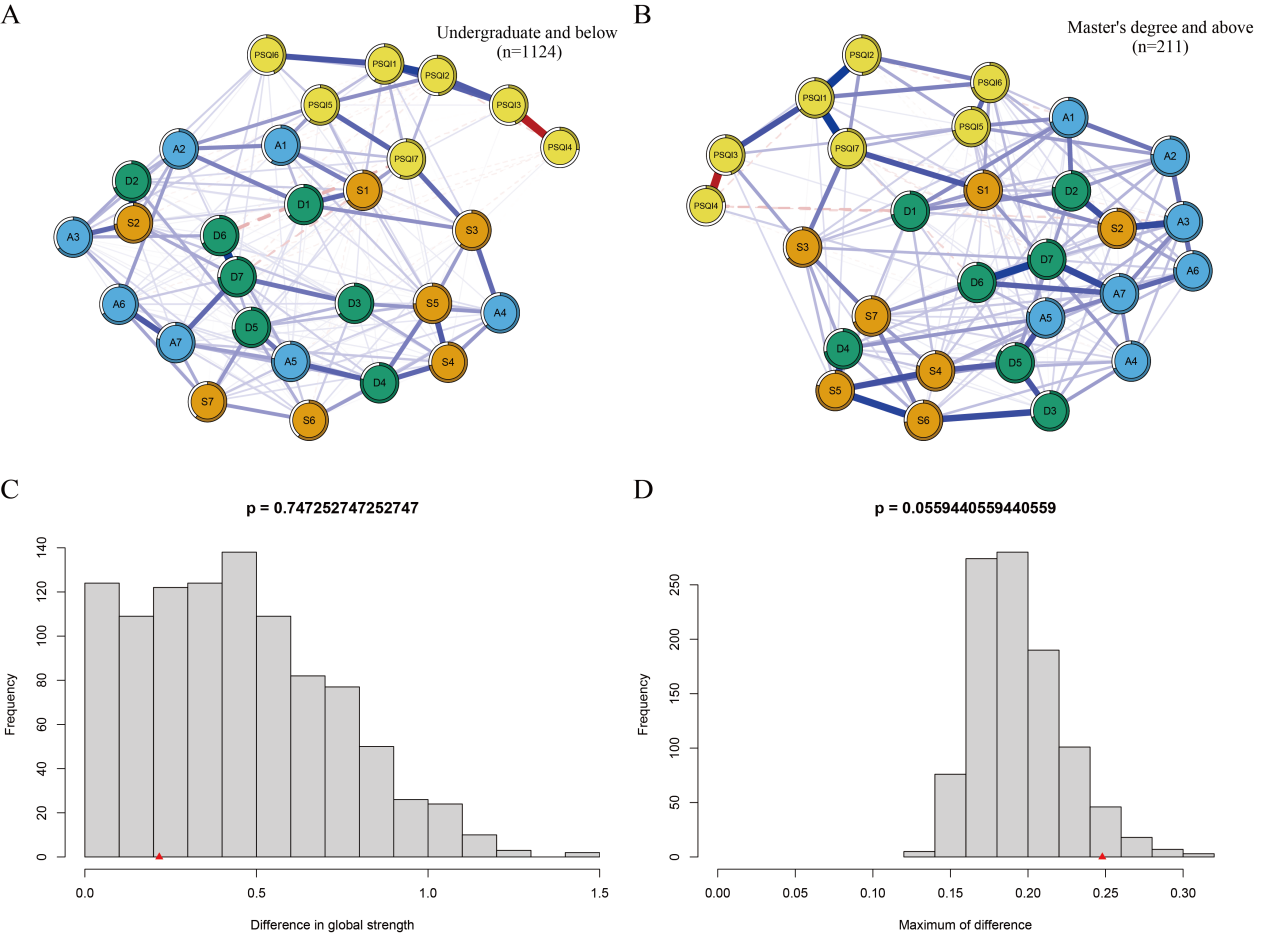


**Figure S9.** Network structure comparison based on education level. (A) Estimated network model for undergraduate and below (n = 1124). (B) Estimated network model for master's degree and above (n = 211). (C) Bootstrap plot illustrating the difference in global strength, which was not statistically significant (global strength for rural: 13.85; for urban: 13.63; p = 0.747). (D) Bootstrap plot of the difference in network structure, showing no significant difference (M = 0.25, p = 0.056). The invariance of edge weights was assessed using permutation tests, yielding p values for each edge comparison. All Bonferroni-Holm adjusted p values were less than 0.05.

**
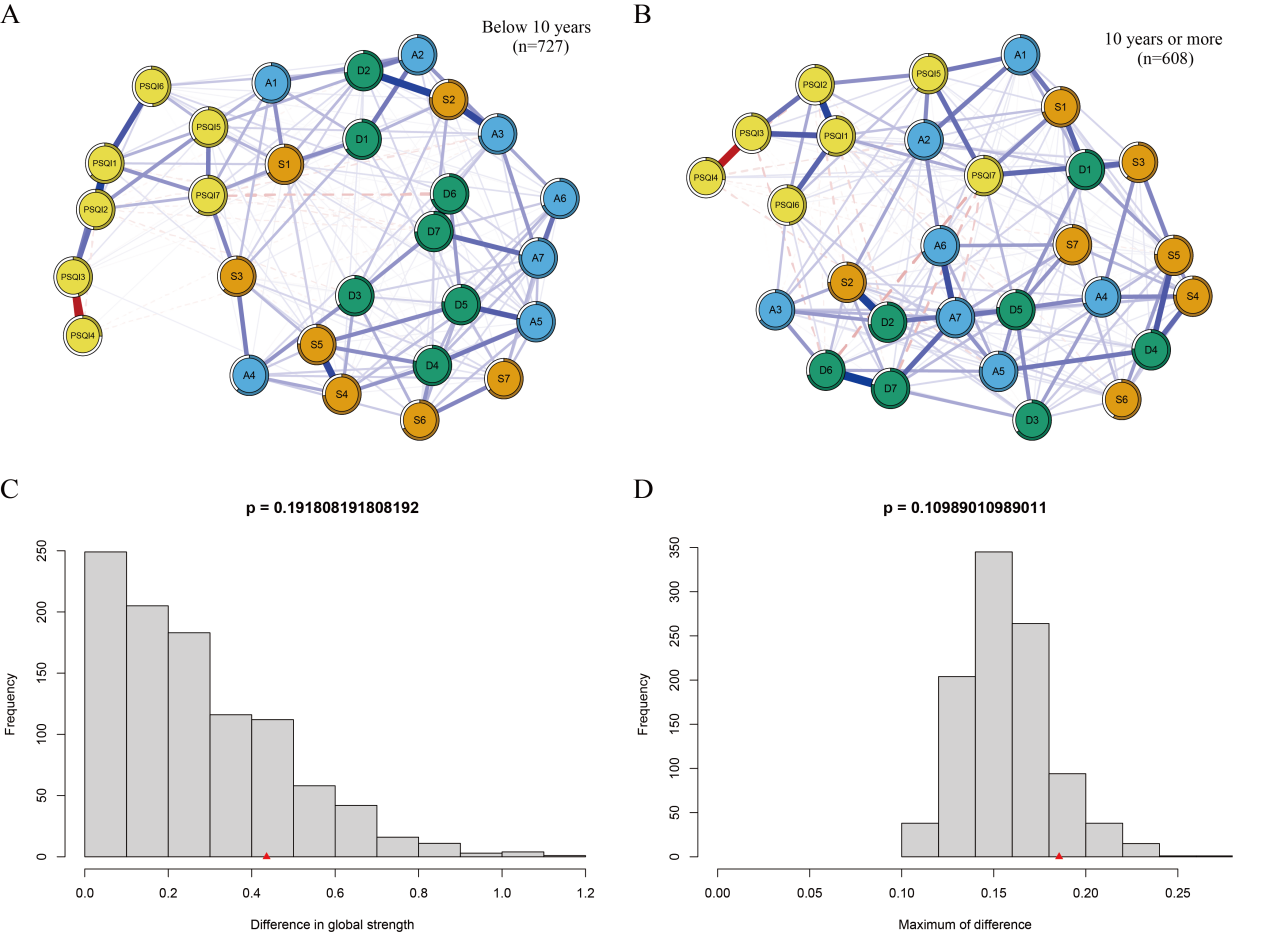
**

**Figure S10.** Network structure comparison based on length of job (years). (A) Estimated network model for below 10 years (n = 727). (B) Estimated network model for 10 years or more (n = 608). (C) Bootstrap plot illustrating the difference in global strength, which was not statistically significant (global strength for below 10 years: 13.86; for 10 years or more: 14.30; p = 0.192). (D) Bootstrap plot of the difference in network structure, showing no significant difference (M = 0.19, p = 0.110). The invariance of edge weights was assessed using permutation tests, yielding p values for each edge comparison. All Bonferroni-Holm adjusted p values were more than 0.05.

**Table S1.** Scales and items used for the assessment of psychopathology symptoms and sleep quality.

| **Scale** | **Abbr** | **Symptoms** | **Items** |
| --- | --- | --- | --- |
| **Stress** | S1 | Upset over little things | 1. I got upset about little things. |
|  | S2 | Overreaction | 6. I found myself over-reacting to situations. |
|  | S3 | Worrying | 8. I was stressing about lots of things. |
|  | S4 | Irritability | 11. I was easily irritated. |
|  | S5 | Difficulty relaxing | 12. I found it difficult to relax. |
|  | S6 | Annoyance with interruptions | 14. I got annoyed when people interrupted me. |
|  | S7 | Easily annoyed | 18. I was easily annoyed. |
| **Anxiety** | A1 | Dizziness | 2. I felt dizzy, like I was about to faint. |
|  | A2 | Breathing difficulties | 4. I had trouble breathing (e.g. fast breathing), even though I wasn't exercising and I was not sick. |
|  | A3 | Shaking | 7. My hands felt shaky. |
|  | A4 | Terrified | 9. I felt terrified. |
|  | A5 | Panic | 15. I felt like I was about to panic. |
|  | A6 | Rapid heartbeat | 19. I could feel my heart beating really fast, even though I hadn't done any hard exercise. |
|  | A7 | Fear without cause | 20. I felt scared for no good reason. |
| **Depression** | D1 | Lack of enjoyment | 3. I did not enjoy anything. |
|  | D2 | Hatred of life | 5. I hated my life. |
|  | D3 | No hope | 10. There was nothing nice I could look forward to. |
|  | D4 | Persistent sadness | 13. I could not stop feeling sad. |
|  | D5 | Self-hatred | 16. I hated myself. |
|  | D6 | Feelings of worthlessness | 17. I felt like I was no good. |
|  | D7 | Terrible life | 21. 1 I felt that life was terrible. |
| **PSQI** | PSQI1 | Sleep quality | 6.In the past month, how would you rate your sleep quality overall. |
|  | PSQI2 | Sleep latency | 2.In the past month, usually wake up at ____ o'clock. 5a.Difficulty falling asleep in the past month (unable to fall asleep within 30 minutes). |
|  | PSQI3 | Sleep duration | 4.In the past month, the actual sleep time per night is usually _____ hours (This may be different than the number of hours you spend in bed). |
|  | PSQI4 | Sleep efficiency | 1.In the past month, the usual time to go to bed at night is ____ o'clock. 3.In the past month, it usually takes ____ minutes from going to bed to falling asleep. 4.In the past month, the actual sleep time per night is usually _____ hours (This may be different than the number of hours you spend in bed). |
|  | PSQI5 | Sleep disorders | 5b.In the past month, wake up in the middle of the night or early morning. 5c.In the past month, go to the bathroom at night. 5d.In the past month, cannot breathe comfortably. 5e.In the past month, cough or snore loudly. 5f.In the past month, feel too cold. 5g.In the past month, feel too hot. 5h.In the past month, nightmares . 5i.In the past month, pain and discomfort. |
|  | PSQI6 | Use of sleep medication | 7.In the past month, how often have you taken medicine (prescribed or “over the counter”) to help you sleep. |
|  | PSQI7 | Daytime Dysfunction | 8.In the past month, Have you often felt drowsy in the past month. 9.Have you had insufficient energy to work in the past month. |

**Table S2.** Fundamental Information on Scales and Item Descriptive Statistics.

| **Items context** | **Mean** | **SD** | **Skewness** | **Kurtosis** | **Strength** | **Bridge strength** | **Predictability** |
| --- | --- | --- | --- | --- | --- | --- | --- |
| S1: Upset over little things | 0.998 | 0.926 | 0.683 | -0.365 | 0.950 | 0.709 | 0.652 |
| S2: Overreaction | 0.625 | 0.873 | 1.229 | 0.499 | 1.107 | 1.032 | 0.755 |
| S3: Worrying | 1.048 | 0.931 | 0.556 | -0.577 | 0.872 | 0.579 | 0.587 |
| S4: Irritability | 0.734 | 0.874 | 1.101 | 0.486 | 1.021 | 0.692 | 0.736 |
| S5: Difficulty relaxing | 0.854 | 0.891 | 0.855 | -0.037 | 1.086 | 0.551 | 0.747 |
| S6: Annoyance with interruptions | 0.678 | 0.863 | 1.173 | 0.593 | 0.813 | 0.541 | 0.622 |
| S7: Easily annoyed | 0.724 | 0.851 | 1.068 | 0.466 | 0.823 | 0.535 | 0.623 |
| A1: Dizziness | 0.971 | 0.946 | 0.642 | -0.574 | 0.813 | 0.557 | 0.578 |
| A2: Breathing difficulties | 0.636 | 0.885 | 1.264 | 0.626 | 0.976 | 0.450 | 0.672 |
| A3: Shaking | 0.523 | 0.823 | 1.513 | 1.411 | 0.897 | 0.527 | 0.680 |
| A4: Terrified | 0.801 | 0.884 | 0.945 | 0.123 | 0.884 | 0.780 | 0.639 |
| A5: Panic | 0.637 | 0.873 | 1.280 | 0.761 | 1.125 | 0.847 | 0.776 |
| A6: Rapid heartbeat | 0.602 | 0.866 | 1.339 | 0.870 | 0.885 | 0.347 | 0.686 |
| A7: Fear without cause | 0.609 | 0.901 | 1.328 | 0.671 | 1.203 | 0.665 | 0.792 |
| D1: Lack of enjoyment | 0.889 | 0.908 | 0.832 | -0.103 | 0.965 | 0.821 | 0.661 |
| D2: Hatred of life | 0.706 | 0.894 | 1.132 | 0.379 | 0.940 | 0.728 | 0.708 |
| D3: No hope | 0.650 | 0.865 | 1.176 | 0.462 | 0.872 | 0.504 | 0.657 |
| D4: Persistent sadness | 0.720 | 0.887 | 1.125 | 0.445 | 1.094 | 0.944 | 0.770 |
| D5: Self-hatred | 0.687 | 0.889 | 1.229 | 0.685 | 1.096 | 0.652 | 0.754 |
| D6: Feelings of worthlessness | 0.400 | 0.771 | 1.907 | 2.711 | 0.913 | 0.428 | 0.687 |
| D7: Terrible life | 0.464 | 0.808 | 1.621 | 1.564 | 1.040 | 0.457 | 0.753 |
| PSQI1: Sleep quality | 1.700 | 0.886 | -0.213 | -0.682 |  |  |  |
| PSQI2: Sleep latency | 1.979 | 0.990 | -0.565 | -0.805 |  |  |  |
| PSQI3: Sleep duration | 1.713 | 0.894 | -0.116 | -0.812 |  |  |  |
| PSQI4: Sleep efficiency | 1.728 | 1.108 | -0.250 | -1.301 |  |  |  |
| PSQI5: Sleep disturbance | 1.577 | 0.821 | 0.220 | -0.635 |  |  |  |
| PSQI6: Use of sleep medication | 0.905 | 1.106 | 0.750 | -0.947 |  |  |  |
| PSQI7: Daytime Dysfunction | 1.758 | 0.994 | -0.359 | -0.912 |  |  |  |

**Table S3.** Bridge Strength linking sleep problem to stress, anxiety, and depression.

|  | **Stress community** | **Anxiety community** | **Depression community** |
| --- | --- | --- | --- |
| **PSQI1** | 0.186 | 0.259 | 0.061 |
| **PSQI2** | 0.103 | 0.166 | 0.176 |
| **PSQI3** | 0.020 | 0.000 | 0.118 |
| **PSQI4** | 0.022 | 0.071 | 0.070 |
| **PSQI5** | 0.428 | 0.498 | 0.369 |
| **PSQI6** | 0.296 | 0.305 | 0.315 |
| **PSQI7** | 0.388 | 0.443 | 0.721 |
